# Movements of Blue Rockfish (*Sebastes mystinus*) off Central California with Comparisons to Similar Species

**DOI:** 10.1371/journal.pone.0098976

**Published:** 2014-06-05

**Authors:** Kristen M. Green, Ashley P. Greenley, Richard M. Starr

**Affiliations:** 1 Alaska Department of Fish and Game, Sitka, Alaska, United States of America; 2 Fishwise, Santa Cruz, California, United States of America; 3 University of California Sea Grant Extension Program, Moss Landing, California, United States of America; California Polytechnic State University, United States of America

## Abstract

Olive (*Sebastes serranoides*), black (*Sebastes melanops*), and blue rockfish (*Sebastes mystinus*) are all common inhabitants of nearshore ecosystems on the West coast of North America and important components of the recreational fishery off California. Acoustic monitoring studies indicate that olive rockfish are highly residential and that black rockfish are capable of long migrations and have less site fidelity; yet little is known about the long-term movements of blue rockfish. External tag-recapture studies indicate that blue rockfish may have intermediate movements relative to these congener nearshore species. To better understand the site fidelity, and daily and seasonal movements of blue rockfish over long (>1-year) time scales, we placed acoustic transmitters into 21 adult blue rockfish (30–41 cm total length) in Carmel Bay, California. Blue rockfish displayed intermediate movement patterns and residency relative to other similar kelp forest rockfish species. Two-thirds of tagged blue rockfish (13 fish) exhibited high residency to the study area (>12 mo). When in residence, mean home range of blue rockfish was 0.23 km^2^, however as many as 30% of tagged blue rockfish shifted their core home range area during the study. Most shifts in home range occurred during upwelling season, and tagged fish moved up to 3.1 km when in residence. Blue rockfish with short residence times were last detected in the study area in late winter and early spring. Blue rockfish were observed at shallower depths during day than night, likely indicative of diurnal feeding. However, over longer time scales, blue rockfish were detected at deeper depths during upwelling periods and with increased wave heights. Daily and seasonal vertical movements of blue rockfish may be influenced by upwelling conditions and local prey abundance.

## Introduction

Blue (*Sebastes mystinus*), olive (*S. serranoides*), and black (*S. melanops*) rockfish are common semi-pelagic nearshore rockfish species in central California that share similar ecological and life history characteristics [1]. All are moderately long-lived (30–50 y maximum life span), mature late (4–9 y of age) and reach similar maximum sizes (53–69 cm) [2]. Black, olive, and blue rockfish typically inhabit similar depths zones in nearshore areas (<100 m), occupy nearshore rock reef and kelp forest habitat, and feed on seasonally abundant prey sources, such as plankton, invertebrates, and young-of-the-year fishes [2], [3]. These three species have historically been characterized as long-term “residents” of these coastal habitats, a hypothesis that is generally supported by external tag-recapture studies [4], [5], [6], [7].

Tag-recapture studies, however, lack detailed information about fish movements. With the advent of acoustic telemetry studies, the paradigm of the “nearshore resident” can be more closely evaluated at daily, seasonal, and annual time scales. For example, although tag-recapture data for olive and black rockfish show relatively similar trends in movement, i.e. in most studies, approximately 90% of fish were recaptured <5 km from release [4], [5], [6], [7], [8], [9], acoustic telemetry studies indicate that these species have distinctly different spatial and temporal movement patterns in the nearshore ecosystem. For example, one study recorded over one-third of black rockfish emigrating from Carmel Bay, California within six months after being tagged; several of these individuals migrated 50–1,000 km north of the study area [10]. In light of this information, a more accurate description for the black rockfish may be a bimodal distribution of movement; i.e. 10 to 40% of individuals migrate, whereas 60 to 90% are residential, depending on the geographic area and age structure of population. In the case of the olive rockfish, however, a long-term acoustic telemetry study corroborated the classic “nearshore resident” hypothesis formed based on external tag-recapture data [11]. In that study, all olive rockfish tagged with acoustic transmitters off Monterey, California showed high site fidelity to the study area, had small mean home ranges (0.00134 km^2^), and utilized even smaller core areas within that home range.

Interpreting the long-term movement patterns for the third species, the blue rockfish, is more difficult in the absence of long-term acoustic telemetry studies. Available data on blue rockfish movements are limited to long-term external tagging studies with poor spatial resolution and one high resolution, but short-term (three week) acoustic study. Tag-recapture studies indicate that blue rockfish may have intermediate spatial movements relative to black and olive rockfish. For example, 90% of tagging studies on blue rockfish have reported a mean recapture distance of <3 km, whereas 10% of blue rockfish movement studies have reported larger movements (as great as 41 km) [7]. Another tagging study suggested that blue rockfish occupy small areas in nearshore reefs in the spring and summer but move to other reefs in the fall and winter [12]. However, unlike the olive and black rockfish, there have been no long-term acoustic telemetry studies of blue rockfish to help interpret these tag-recapture trends. To date, only one acoustic telemetry study on blue rockfish has been published [13]. That study reported limited home ranges (0.00878 km^2^) for 10 acoustically tagged blue rockfish in northern California, yet the short duration of the study (21 d) and small spatial scale precluded any conclusions regarding seasonal and annual patterns of movements. From these studies, it seems probable that blue rockfish maintain small home ranges for at least part of the year, but whether these home ranges expand or if migration occurs over longer time scales has yet to be determined.

We designed a study to track the movements of adult blue rockfish over a 15-month period using acoustic telemetry in Carmel Bay, California, USA. Our goal was to explore the movement patterns of blue rockfish and determine whether movements of blue rockfish fit the paradigm of the nearshore resident (e.g. olive rockfish), or are characterized by greater seasonal movements that are related to environmental conditions. The specific objectives of this study were to: (1) quantify annual home range and residency of blue rockfish, (2) describe the daily and seasonal movement patterns of blue rockfish with respect to local oceanographic conditions; and (3) estimate factors influencing detection capabilities of acoustic receivers in nearshore physical environments.

## Materials and Methods

### Ethics Statement

This study was conducted with the approval of San Jose State University, and carried out in strict accordance with the San Jose State University Animal Welfare Policy according to Academic Senate Policy F06-4. The protocol was approved by the Institutional Animal Care and Use Committee (Permit Number: 824). All surgery was performed under tricaine methanesulfonate (MS 222), and all efforts were made to minimize suffering. This research was conducted under California Department of Fish and Game Scientific Collecting Permit SC-008142.

### Study Site

**Figure 1:**
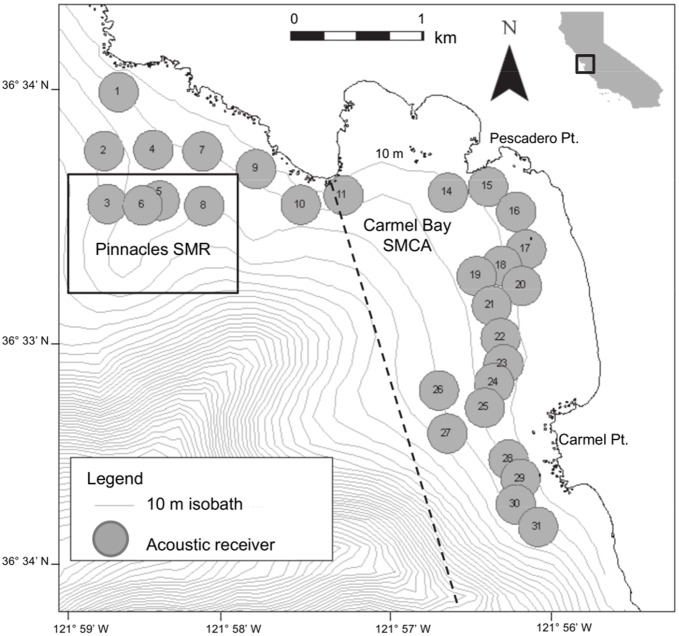
Study location in Carmel Bay with Marine Protected Area boundaries and receiver locations. Dashed line indicates the State Marine Conservation Area (SMCA). Solid line surrounds the State Marine Reserve (SMR). Numbered grey circles represent receiver locations with estimated 150 m radius. Depth contours are based on 10 m isobaths.

The study site was located in a 4 km by 2 km area located within Carmel Bay, California (Figure 1). Topography in Carmel Bay includes high-relief rock areas and sand flats interspersed with low rocks. The high-relief areas are comprised of large boulders and rock outcrops up to 5 m in height. Sand areas with scattered rocks contain boulders with 1–2 m of vertical relief. Seasonal kelp beds of *Macrocystis pyrifera*, *Nereocystis luetkeana* and perennial understory algae *Pterygophera californica* are abundant in the rocky habitats. The majority of the study area is within the boundaries of two Marine Protected Areas (MPAs) (Figure 1). The Carmel Bay State Marine Conservation Area (SMCA) allows recreational fishing but prohibits commercial fishing. The Carmel Pinnacles State Marine Reserve (SMR) prohibits take of all marine species.

### Fishing, Tagging, and Data Collection

We caught blue rockfish with rod and reel fishing gear during September and October 2006. To avoid damage due to barotrauma, we targeted and caught fish in <15 m of water. Large blue rockfish (> length at 50% maturity) [14] were selected for tagging if they showed no symptoms of barotraumas. Sex ratio of tagged blue rockfish (assessed based on presence or absence of urogenital papillae) were similar, but we did not target males or females. Captured blue rockfish were anesthetized in a solution (0.1 g L^−1^) of MS 222 and Vemco V13P-1H-S256 acoustic transmitters (Vemco Ltd., Nova Scotia, Canada) were surgically implanted into the peritoneal cavity. Transmitters emitted coded signals, which included fish depth, at a random interval between 90–270 s. Predicted battery life for each transmitter was 445 d. An external T-bar anchor tag imprinted with a unique tag identification number also was implanted into the dorsal musculature of the fish in case of angler recapture. Fish were released at the location of capture.

We placed 29 acoustic receivers (Vemco VR-2) along the coastline between Pescadero Point and Carmel Point, in depths of 7–40 m (Figure 1). Receivers were individually anchored to a 40 kg mooring weight using a line that extended approximately 5 m above the sea floor. A subsurface float was attached on a line above the receiver to maintain line tension and keep the receiver upright in the water column. Temperature loggers (Onset StowAway Tidbit Underwater Data Loggers, programmed to record water temperature every 15 min) were attached on the mooring line of four receivers, spread throughout the array. Temperature loggers on those mooring lines were positioned at shallow (12–16 m), mid (18–24 m), and deep (26–34 m) depth intervals. Data from moored receivers and temperature loggers were downloaded every 4–6 mo between September 2006 and January 2008.

Acoustic detection capabilities of VR-2 receivers are affected by biological and anthropogenic noise, sea state, bottom topography, and submerged vegetation [15]. To estimate VR-2 detection ranges in Carmel Bay, we performed range-testing trials in the kelp forest before the array of receivers was deployed for this study. Range testing was conducted in late summer of 2005, during peak summer kelp densities, to gain a conservative estimate of detection ranges in dense vegetation. For the range tests, a V13 transmitter was attached to a weighted line and deployed at 50 m intervals from receivers stationed throughout the kelp bed. The transmitter was suspended 1 m from the seafloor and was held at each station for 15 minutes. The receivers were collected and brought to the lab for data download. We determined the number of signals recorded in each 15-minute period and calculated the percentage of signals recorded relative to the number of signals transmitted. From these trials we determined that a signal transmission had >80% chance of being recorded when the tag was within a range of 150 m. Based on these range estimates, the array was configured with approximately 300 m spacing between receivers so as to minimize the overlap of detection ranges among receivers.

The variation in receiver detection ranges over longer time scales was examined by deploying a reference transmitter (Vemco V13-1H-RO4K) 1 m off the seafloor at a fixed location, 140 m equidistant from two receivers located near kelp beds. Reference transmitter data were then correlated with physical parameters including time of day, wave height, mean wave direction, and wind speed. Differences between day and night signal detection for the reference pinger were compared using different filtering methods and tested using a Pearson’s chi-square test. The reference transmitter was deployed from March through September of 2007.

Oceanographic and atmospheric data were acquired from the historical data archives of the National Oceanic Atmospheric and Administration (NOAA). Wave height, wind speed, and barometric pressure were recorded from Monterey Buoy 46042, (http://www.ndbc.noaa.gov). Day lengths were derived from the historical archives of the US Naval Observatory from Carmel, California (http://aa.usno.navy.mil/data/). Daily and monthly upwelling indices, expressed as m^3 ^s^–1^ 100 m^–1^ of coastline at 122° W, 36° N, were obtained from NOAA Fisheries Pacific Fisheries Environmental Laboratory (PFEL) (www.pfeg.noaa.gov/products/PFEL).

### Data Analyses

Residence times for all tagged blue rockfish were calculated by dividing the total number of days a fish was present in the array by the predicted battery life of the transmitter. Daily presence in the array was defined as ≥2 signal detections in a 24 h period. The filtering method of ≥2 signal detections in a 24 h period was based on range testing data from a stationary reference transmitter in the study area as well as a review of the literature; however, the vast majority of tagged fish in our study had >100 detections each day. The percentage of days each fish was absent from the array was calculated as the number of days absent divided by the number of days from release to the day the last tag transmission was recorded (within transmitter’s predicted battery life). Occasionally, receivers were temporarily missing from the array because they were lost in storm events. For presence and absence calculations for a given fish, we excluded the dates from analyses for which a receiver was missing due to storms.

Diel changes in presence of blue rockfish were characterized by calculating the percentage of hours a fish was detected in the study area during the day versus at night. Day hours were defined as the time period between sunrise and sunset. Night hours were defined as the time from 1 h after sunset until 1 h before sunrise. Crepuscular hours (1 h before sunrise and 1 h after sunset) were excluded from analyses. A fish was considered to be present in an hour if there were ≥2 tag detections in that hour. The number of hours a tagged fish was detected during the day or night on each date was normalized by the total number of night or day hours available on that date and summed to create a contingency table with the expected and observed percentage of hours detected for night periods versus day periods. Fisher’s exact test was used to test the null hypothesis that there was no difference in fish presence in the study area, either between day and night periods annually or for selected seasons.

Vertical movements of blue rockfish were examined by comparing differences in depth anomalies of tagged fish. To calculate daily depth anomalies, we subtracted the annual mean depth of a given fish from the daily mean depth of that fish on each date of the study. Depth anomalies were averaged across all fish on each date of the study to obtain a mean anomaly for each date and for day and night periods. Positive anomalies represent deeper mean depths than the annual mean depth; negative anomalies represent shallower mean depths than the annual mean depth.

We used cross correlation analysis and a generalized linear model (GLM) to compare daily depth anomalies of blue rockfish with water temperatures, the square of mean wave heights, wind speeds, upwelling indices, and atmospheric pressure [16]. Cross correlation analysis removes the effect of autocorrelation inherent in time series data by fitting univariate models to the *X* and *Y* time series, using these fitted models to obtain estimated residuals, and then determining the cross correlation function between the two residual time series. We conducted the cross correlation analysis for a year period (October 2006–October 2007), the high upwelling season (April–June), and the low upwelling season (December–February), as defined by monthly upwelling indices. To compare mean day depths with mean night depths of fish across the different oceanographic seasons, we used a repeated-measures ANOVA implemented through a mixed-effect model with depth as the dependent variable and fish as the random term. In the model, we tested a time of day effect, a seasonal effect, and an interaction term.

We calculated a measure of home range for each tagged fish in the following manner. For each hour that a fish was present in the array, it was assigned one or more locations, based on the receiver(s) that recorded signals from the tagged fish. The number of hours detected at each receiver location was tallied for each fish. Receiver locations that ranked in the 90^th^ percentile of the total hours that a fish was recorded during time at liberty were used to calculate the home range of that fish. In ArcGIS, a polygon with the estimated home range for each individual fish was drawn around these receiver locations, assuming the full receiver detection radius of 150 m from the receiver location. The area encompassing home range for each fish was calculated using the ArcGIS spatial analyst extension, XToolsPro.

## Results

### Fishing and Tagging

Twenty-one blue rockfish were captured, tagged, and released in the study area between 27 September and 11 October 2006 (Table 1). Fourteen of 21 fish were above the length at 100% maturity, and all blue rockfish were above the length at 50% maturity [14]. Mean total length of the 21 blue rockfish was 33.7 cm±0.7 (mean ± SE). One fish (Tag 11) was only detected in the study area for 13 d. As there were no complications for this fish during surgical procedures, and this fish was observed swimming upon release, one possibility is that the transmitter malfunctioned. Depth data were not transmitted for this fish, which may be another indication of transmitter failure. This fish was excluded from all analyses.

**Table 1:** *Sebastes mystinus* tagged and released in the study area in Carmel Bay, California.

ID	TL (cm)	Release Date	Total d det.	Total d avail.	Res. Time (% of d)
8	31	9/27/2006	199	445	44.7
9	35	9/4/2006	425	445	95.5
10	30	9/27/2006	435	445	97.8
11	33	9/27/2006	13	445	2.9
12	41	9/27/2006	415	445	93.3
13	31	9/27/2006	440	445	98.9
14	34	9/27/2006	312	415*	75.2
15	32	9/27/2006	376	380*	98.9
16	34	9/27/2006	445	445	100.0
24	35	9/28/2006	401	445	90.1
25	30	10/11/2006	381	383*	99.5
26	30	10/5/2006	406	445	91.2
27	35	10/5/2006	364	365*	99.7
28	38	9/29/2006	193	445	43.4
29	34	10/3/2006	178	419*	42.5
30	31	10/5/2006	144	445	32.4
31	35	10/5/2006	237	445	53.3
32	37	10/5/2006	368	368*	100.0
33	36	10/5/2006	441	443*	99.5
34	35	10/5/2006	219	445	49.2
35	30	10/11/2006	368	368*	100.0

Tag number (ID), total length (TL), and release date of blue rockfish in the study area. Total days detected (Total d det.) is the number of days in which ≥2 signals were recorded in a 24 h period for an individual blue rockfish. Total days available (Total d avail.) is the number of days possible for each fish to be detected after receiver loss was accounted for. Residence time (Res. Time (% of d)) is the total number of days detected for each fish divided by the total number of days available in the battery life of a transmitter. Asterisks indicate individuals where residence time was adjusted for dates when receivers were temporarily lost in storms.

### Range Testing and Reference Transmitter

Preliminary range trials, conducted before the tagging study began, enabled us to estimate VR-2 detection ranges of 150 m in nearshore areas near kelp forests. Long-term acoustic testing with the stationary reference transmitter, placed 140 m equidistant between two receivers, revealed that the total number of daily detections received was highly variable, ranging from 7–401 detections per day. An average of 9.3±5.9 signals/h was recorded from the reference transmitter during the day and 6.1±4.3 signals/h at night; this difference was significant (Pearson’s chi-square = 9.667; p = 0.022). When daily (≥2 signals/d) and hourly (≥2 signal/h) detection filters were applied to the data, the reference transmitter was detected for 100% of the days and 91% of the nights it was deployed.

We found no significant (p>0.05) correlations among detections of the reference transmitter and wave height, mean wave direction, or wind speed. Thermal stratification at deeper depths did cause variation in reference transmitter detections. A positive correlation (r^2^ = 0.5878, p<0.05) was found between the number of hours without recorded signals and Δ T, when the temperature differential between 14 m and 31 m was >1°C.

### Residence Times

**Figure 2:**
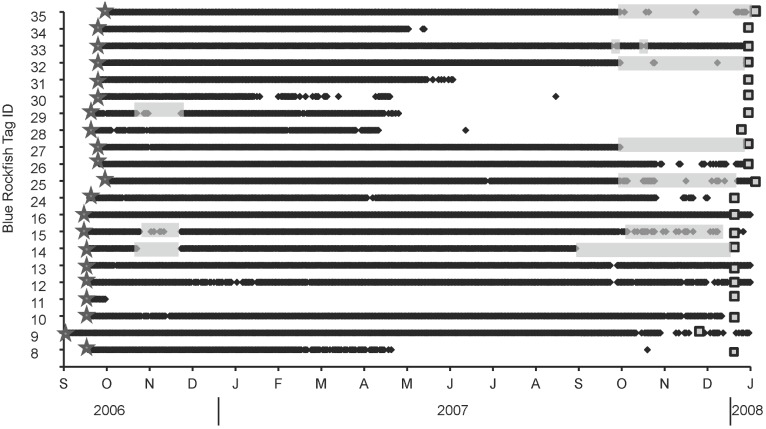
Residence times of blue rockfish in Carmel Bay, CA. Tag number of each blue rockfish is listed on y-axis. Black diamonds represent detection of signals from a tagged fish. Stars depict date of release of each fish, grey squares indicate projected battery life of each tag, and transparent grey bars represent time periods during which a receiver that was within the home range of a tagged fish was missing from the array due to storms.

Residence times of blue rockfish in the study area ranged from 32.4–100% (144–445 d) of the expected battery life of transmitters (Figure 2). Mean residence time of blue rockfish was 337.4 d±22.8). Thirteen of 20 individuals were detected on >364 out of 445 days at liberty. The remaining seven blue rockfish were present in the study area for a mean of 48.7%±5.1 of the days of transmitter battery life, but even these fish with lower residence times were in the array for >6 mo before permanent departure. We could detect no relationship between residence time and total fish length using linear regression (p = 0.936).

### Absences

Five blue rockfish were present in the study area 100% of days during time at liberty after receiver loss was accounted for. Of the remaining 15 fish that did periodically leave the study area, absences were <15% of the total time at liberty. There was no daily pattern of synchronous absences and returns from the study area among fish, although the six fish with the shortest residence times were last detected in the array during the upwelling season (between late April and June 2007). Four of these fish were last detected within the two-week period between 18 April and 2 May 2007, at the peak of the spring upwelling season. Also, absences were recorded for eight fish for 1–3 d during intense storms (4 December 2007 and 5 January 2008), when sea states exceeded 10 m for >24 h.

### Diel Movements

There was no difference in the proportion of hours blue rockfish were detected in the study area between day and night periods (Pearson’s chi-square = 13.144; p = 0.069), nor for the high and low upwelling seasons (April–June, Pearson’s chi-square = 8.60; p = 0.283; December–February, Pearson’s chi-square = 11.39; p = 0.133). Blue rockfish were detected for an annual mean of 77.7%±3.9 of day hours and 59.5%±5.0 of night hours. The monthly mean percentage of hours in which tagged fish were detected during the day was relatively constant (76–90%) between October 2006 and October 2007. Monthly mean percentage of night hours with detections was consistently lower than the mean percentage of daytime hours (49–68% of night hours). The highest mean nighttime presence occurred in August 2007 (68.3%±7.4).

### Vertical Movements

**Figure 3:**
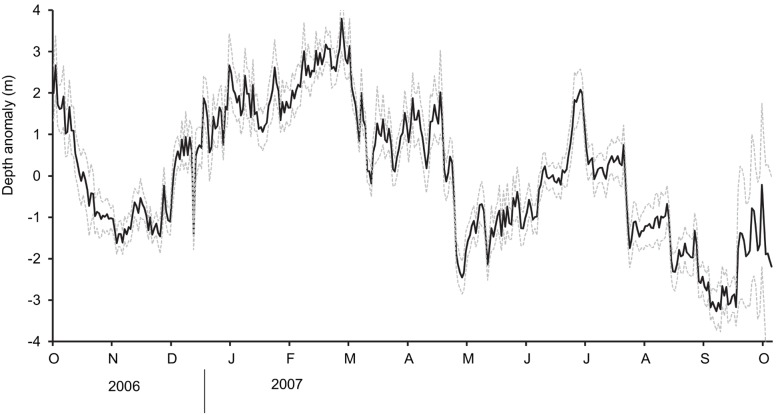
Mean daily depth anomaly for blue rockfish. Mean daily depth anomaly (solid black line) for 20 blue rockfish from 1 October 2006 through 30 September 2007. Grey dashed lines above and below the solid black line indicate ± SE. A daily depth anomaly was obtained by subtracting the annual mean depth of a given fish from the daily mean depth of that fish on each date of the study. Mean daily depth anomalies were obtained by averaging the depth anomaly for all fish on each date. Positive anomalies represent deeper mean depth than the annual mean depth; negative anomalies represent shallower mean depth than the annual mean depth.

Water depths in the study area ranged from 10–20 m, and tagged blue rockfish were released throughout the study area in depths between 5 and 12 m. Tagged fish often exhibited depth changes of 5–10 m over short time intervals (5–20 min). The mean annual depth of all blue rockfish was 8.7 m±0.6; mean annual depths of individuals ranged from 5.5–12.6 m. An evaluation of blue rockfish depths using a mixed-effects model showed significant interactions between depth and time of day (i.e. day/night) and season (p<0.0001), with no significant interaction between season and time of day (Table 2). Tagged fish were deeper at night than during the day and deeper during the low upwelling (winter) than high upwelling (spring) season. Over a year period, the mean daily depth anomaly for blue rockfish fluctuated seasonally (Figure 3).

**Table 2:** Mixed model, least-squares means estimates for depths of tagged blue rockfish in Carmel Bay in different seasons and times of day.

Effect	TOD	Season	Depth (m)	SE	DF	t Value	Pr > |t|
TOD	day		8.8	0.06	19	152.54	<.0001
TOD	night		10.1	0.07	19	152.76	<.0001
season		low upwelling	9.11	0.05	57	175.44	<.0001
season		high upwelling	8.9	0.08	57	115.74	<.0001

TOD: time of day, i.e. day or night. Seasons are defined as low upwelling (December–February) and high upwelling (April–June).

### Environmental Correlations

**Figure 4:**
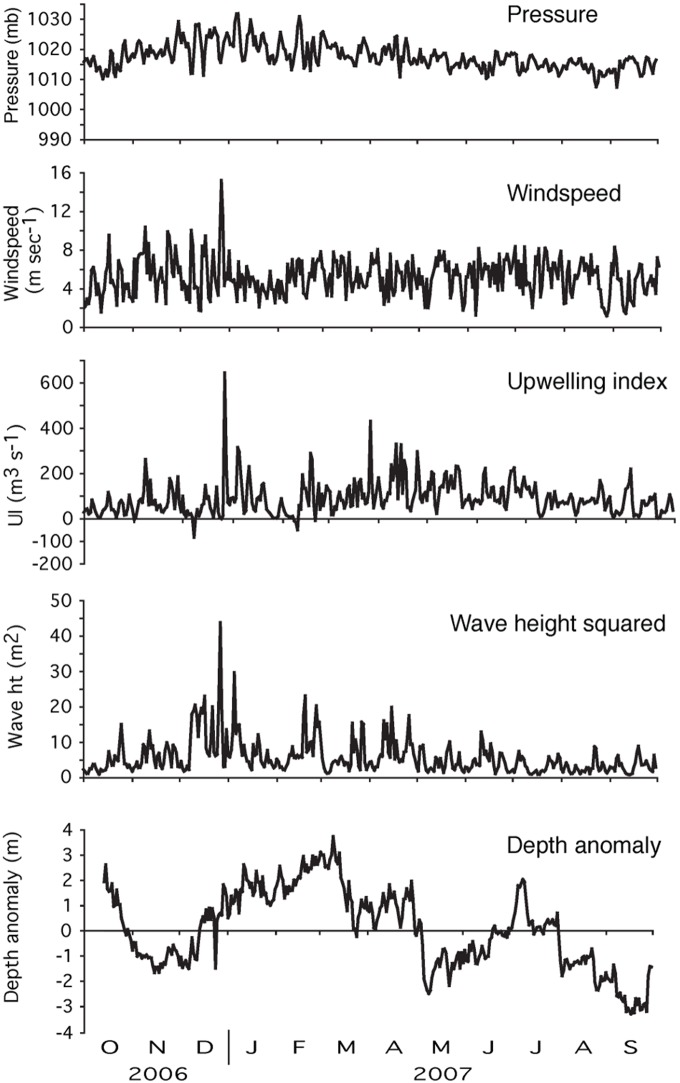
Time series plots of environmental variables and blue rockfish depth anomalies. Plots of mean daily barometric pressure (mb), wind speed (m sec^−1^), upwelling index (m^3 ^s^−1^ 100 m^−1^ of coastline), mean wave height (m^2^), and depth anomalies (m) of tagged blue rockfish. A daily depth anomaly was obtained by subtracting the annual mean depth of a given fish from the daily mean depth of that fish on each date of the study. Positive anomalies represent deeper mean depth than the annual mean depth; negative anomalies represent shallower mean depth than the annual mean depth.

The GLM analysis indicated significant positive relationships between the depth anomaly and upwelling indices, wave height, and atmospheric pressure (p<0.001). Cross correlation analyses of all data combined indicated that depth anomalies of tagged blue rockfish were positively correlated with upwelling indices with a lag of 1 d (p = 0.001), atmospheric pressure with no lag (p = 0.030) and the square of wave height (p<0.001) with a lag of 1 d (Table 3; Figure 4). There were weak but significant negative correlations between temperature at all depths (17, 29, and 31 m) and depth anomalies (p = 0.001; p = 0.020, and p = 0.008 respectively) with no lag (Table 3). Positive correlations indicated that tagged fish moved deeper when the upwelling index, atmospheric pressure, and wave height (squared) increased. Also, fish moved deeper with declining temperatures.

**Table 3:** Lag in days, correlation, and p values associated with cross correlations among selected environmental variables and the depth anomalies of tagged blue rockfish for the periods 16 October 2006 to 15 October 2007, 1 December 2006 to 28 February 2007, and 1 April 2007 to 30 June 2007.

Time Period	Variable	Lag time (d)	Correlation	p
10/16/06–10/15/07	Upwelling index	1	0.20	0.001
	Atmospheric pressure	0	0.11	0.030
	Wave height (squared)	1	0.18	<0.001
	Temperature (17 m)	0	−0.17	0.001
	Temperature (29 m)	0	−0.13	0.020
	Temperature (31 m)	0	−0.14	0.008
12/1/06–2/28/07	Upwelling index	1	0.28	0.007
	Atmospheric pressure	0	0.32	0.002
	Wave height (squared)	1	0.27	0.009
	Temperature (17 m)	0	−0.49	<0.001
	Temperature (29 m)	0	−0.47	<0.001
	Temperature (31 m)	0	−0.44	<0.001
4/1/07–6/30/07	Upwelling index	1	0.33	<0.001
	Atmospheric pressure	2	0.21	0.050
	Wave height (squared)	0	0.26	0.014
	Temperature (17 m)			ns
	Temperature (29 m)	1	−0.21	0.044
	Temperature (31 m)	0	−0.22	0.040

Positive correlations indicate that tagged fish move deeper when upwelling index, atmospheric pressure, or wave height (squared) increased. Tagged fish moved deeper when temperatures declined (ns = not significant, p>0.05). Temperature was sampled at three water depths: 17, 29, and 31 m.

When evaluated with respect to the months with the highest mean monthly upwelling indices (April–June), depth anomalies were again positively correlated with upwelling indices (p<0.001), atmospheric pressure (p = 0.050), and the square of wave height (p = 0.014), all with lags of 0–2 d (Table 3; Figure 4). There was no significant relationship between blue rockfish depth anomaly and water temperature at 17 m, but there were significant negative correlations at 29 m (p = 0.044) with a lag of 1 d and at 31 m with no lag (p = 0.040) (Table 3). During the winter season (December–February), there was a significant positive correlation between depth anomaly and upwelling (p = 0.007), atmospheric pressure (p = 0.002), and the square of wave height (p = 0.009) with lags of 0–1 d (Table 3; Figure 4). There was a strong negative correlation between depth and temperature at all depths (p<0.001) (Table 3).

### Home Range

**Figure 5:**
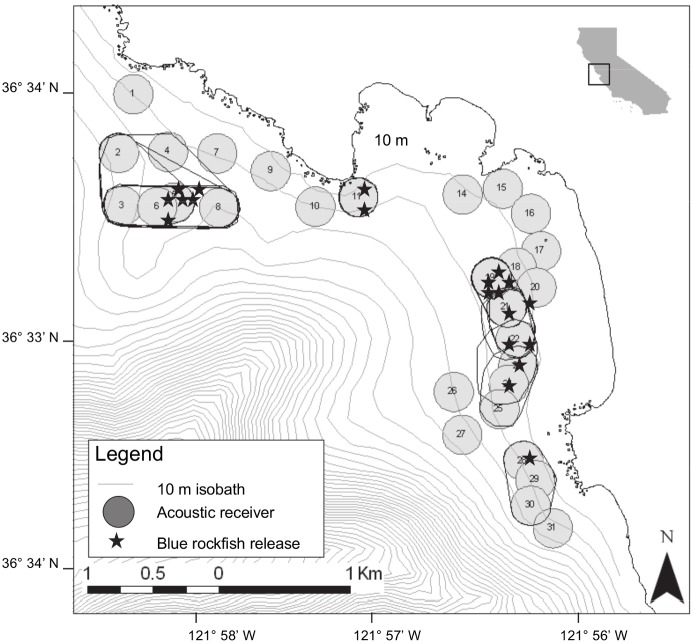
Release locations and estimated home range areas for tagged blue rockfish in the study area. Numbered grey circles represent receiver locations with estimated 150% of the h at liberty.

Home range areas for blue rockfish ranged from 0.07–0.53 km^2^. Mean home range area was 0.23 km^2^±0.03, and a mean of 2.8±0.3 receiver locations were used in these home range estimations. Regression analysis revealed no significant relationship between total length and home range areas for all fish combined (p = 0.329). Home ranges of blue rockfish were centered near the original site of release for each fish (Figure 5), but two fish later shifted to a different location and took up a new home range during the course of the study. Of the two fish that exhibited a shift in home range during their time at liberty, one (Tag 34) was released near Receiver 19, and detected in that area for seven months until it moved 1.5 km to Pescadero Point (near Receivers 10 and 11) (Figure 5). The other fish, (Tag 26) was detected near Receiver 21 for 380 consecutive d; but moved 3.1 km north to the Pinnacles SMR, and then traveled to Pescadero Point (Figure 5). The blue rockfish carrying Tag 26 was detected sporadically in this area until 3 January 2008, when the transmitter battery likely expired. Shifts in home range for these tagged fish occurred near the end of their time at liberty in the study area, and thus these new locations did not rank within the 90^th^ percentile of the total hours that a fish was recorded during time at liberty and were not included in the home range calculations.

## Discussion

### Residence Time and Absences from the Array

As predicted, blue rockfish displayed a fidelity to the study area that was higher than reported for black rockfish [10] but less than for olive rockfish [11]. Whereas the majority of tagged blue rockfish (65%) were present in the study area for over 90% of time at liberty, six fish (30%) had residence times of only 4–8 mo. However, of those fish with short residence times, four individuals were detected intermittently on other receivers in the study area. The detections for each of these fish occurred on five to fifteen occasions, but only as a single detection in a 24 h period, and thus did not meet the filtering criteria for the residency analyses. These detections may be spurious. However, we saw small home range shifts of two fish inside the study area, thus we believe that these fish with intermittent detections may also have moved to a new home site just outside the range of receiver detection. This represents an interesting situation with respect to how residency is interpreted when considering the design of marine reserves. Several authors have suggested that marine reserves sizes be based on the home range size of protected species [17], [18], [19], [20]. If protected fish in a marine reserve, however, periodically shift their home range location from the reserve to a new site, then reserve protection is greatly reduced, even if home range size remains the same. Thus, basing management on average home ranges can be problematic by excluding the variability in behavior expressed by different species [21]. Longer-term acoustic studies are needed to determine how frequently individual rockfish shift from one home site to another over the course of their lifetime. Longer-term studies can also inform MPA modelers about the shape of dispersal curves; e.g., whether dispersal is linear over time or occurs at irregular frequencies, and also can provide insights about the proportion of the population that undertakes a shift in home range over time.

Although blue rockfish displayed lower site fidelity during the upwelling season, winter storms did not affect residency of blue rockfish. In a similar study in Carmel Bay, black rockfish emigration was highly correlated with major storm events [10]. The blue rockfish in our study were present for the same intense storms in Carmel Bay that coincided with the departure of over 40% of small adult black rockfish, but unlike black rockfish, blue rockfish emigration did not coincide with storm dates. We did observe, however, that the majority of blue rockfish that were present in the study area during these winter storms went undetected for 24–72 hours during major storms. Cross correlation analyses indicated that tagged blue rockfish moved deeper with increasing wave heights and these fish may have sought shelter in rocks or crevices during storms, thus preventing detections during these turbulent periods. Similar behavior for blue rockfish has been documented in other areas during winter storms [22]. In addition, unlike tagged small adult black rockfish [10], all the blue rockfish tagged in this study were above length at 50% maturity, and most were above lengths at 100% maturity. Some studies indicate that older fish are better able to maintain site fidelity than younger fish [3], [23] so these older blue rockfish may have been better equipped to maintain position in the study area than black rockfish. Similarly, the mature olive rockfish tagged in Monterey, CA demonstrated high site fidelity regardless of upwelling season or winter storm height [11]. In a different acoustic study, vermilion and copper rockfish were described to demonstrate a combination of high site fidelity with occasional shifts in home ranges, while the rockfish that displayed greater movements were all below the length at 50% maturity [24].

### Home Ranges

Published studies have reported that blue rockfish have small home ranges [7], [13], [22], the smallest of which (0.009 km^2^) was obtained from a short-term, small-scale acoustic positioning study [13]. Our study covered a large extent (>4 km) of coastline within Carmel Bay, CA, rather than a small area with a high density of overlapping receiver detection ranges, so that we could provide an understanding of longer-term movements. We were, however, able to utilize the expected reception distances of the receivers to estimate blue rockfish home ranges for a 15-month time period. The mean home range in our study was at least 25 times larger than the prior acoustic study on blue rockfish [13]. This underscores the need for the use of studies with longer time frames to describe fish movements for resource management purposes.

One of our hypotheses was that blue rockfish would have a smaller home range than the more mobile, piscivorous black rockfish. However, blue and black rockfish mean home ranges, were nearly equal in Carmel Bay, CA (0.23 km^2^ and 0.25 km^2^ respectively). Similar to black rockfish [10], as many as one-third of the tagged blue rockfish in Carmel Bay moved out of the acoustic array during the study time period. However, black rockfish migrated long distances northward, whereas blue rockfish appeared to move just several kilometers to a new location within Carmel Bay. After this moderate shift in location, blue rockfish appeared to maintain a small home range. Two blue rockfish migrated to a different location within the study area after relatively long site fidelity to one location (seven and twelve months, respectively).

Our hypothesis that blue rockfish would have larger home ranges than olive rockfish proved to be true. The mean home range of blue rockfish in our study was several orders of magnitude larger than that reported for olive rockfish (0.0013 km^2^) [11]. Similar to blue rockfish, olive rockfish were active during daylight hours and moved less at night in Monterey, CA [11]. Tagged olive rockfish also were continuously present throughout the year long tracking study, corroborating the paradigm of olive rockfish as a nearshore resident [11]. However, due to the limitations of transmitter battery life when continuously tracking fish, olive rockfish were tagged in three batches (spring, summer, winter) and tracked for four months (duration of battery life) each season. Based on the seasonal movements of individual blue rockfish we tagged, it is likely that a larger home range for olive rockfish would have been reported had individual fish been tracked for an entire year.

Blue rockfishes are reliant upon local oceanographic conditions for food and demonstrate immediate feeding responses to increased zooplankton densities in northern California [25], [26], [27], [28] during periods of intermittent relaxation, when zooplankton and fish larvae are transported onshore from offshore upwelling centers [29], [30]. In our study, this reliance on zooplankton densities manifested itself in the seasonal contraction and enlargement of blue rockfish home ranges. Most blue rockfish with short residence times were last observed in the study area during the spring, when food availability may have been dependent on upwelling events, and kelp biomass (shelter for blue rockfish) was at a minimum. We hypothesize that at least two blue rockfish that made distinct shifts in activity moved in search of prey or to stay within water masses that contained prey. However, the trends in seasonal home ranges for olive and black rockfish differed from blue rockfish; the mean home range of olive rockfish was significantly greater in the fall than the rest of the year [11]. In the summer in Carmel Bay, black rockfish stay in the kelp beds where there are high densities of young of the year rockfish prey, but the rest of the year they expand their home range offshore during the day [10]. Unlike blue rockfish, black and olive rockfish are primarily piscivorous, but will switch to eating zooplankton during the non-upwelling seasons [3], [26]. For longer-term studies, we would expect blue rockfish to maintain small home ranges for months at a time, but inevitably migrate to new areas cued by a need for food, shelter, or mating, and then maintain a small home range until stimulated to shift again.

### Diel and Environmental Movements

Diel movements of rockfishes have been reported for a variety of rockfish species [21], [24], [31], [32], [33]. Blue rockfish in our study exhibited diurnal vertical movement patterns throughout all seasons of the year. Blue rockfish in our study were shallower during the day than at night and made larger vertical movements during day than at night. Our observations were consistent with SCUBA surveys, in which blue rockfish were present in higher densities during the day; presumably to feed in the water column, but sheltered in holes on the seafloor at night [34]. This supports conclusions from previous studies that blue rockfish are diurnal predators based on greater spatial movements during the day [3], [13], [26].

Similar to previous studies [3], [13], we found that blue rockfish movements were correlated with local environmental variation. In our study, blue rockfish moved deeper with increased upwelling, decreased water temperature, and an increase in wave energy. Lag times were typically 0.5–2 d. Our speculation about causes for these movements is similar to reasoning proposed in a prior study [3]; we believe that during seasonal periods of upwelling, blue rockfish are moving to follow local water masses that contain high densities of plankton. The tendency of blue rockfish in our study to move deeper with increased wave height may be a sheltering mechanism to maintain position in the area during powerful storms with strong currents.

Although blue, olive, and black rockfish occupy the same general spatial areas annually, the vertical distributions of the three species were significantly different from one another during the non-upwelling season [3]. During the upwelling season, densities of blue rockfish have been documented to increase with depth relative to the non-upwelling season [3], black rockfish move deeper with upwelling events [10], while most of the year olive rockfish maintain small home ranges within the kelp bed [11].

### Acoustic Receiver Detection Range

Many acoustic studies have been conducted and published without measurement or description of the acoustic underwater environment and how it affects the detection range of receivers. Characterizing the acoustic properties of the water column and how they affect the transmission of animal transmitter signals is critical to prevent false conclusions about movements of tagged animals that may actually be artifacts of the underwater environment. In our study, a reference transmitter was recorded significantly more times during the day than at night. This was likely due to the interference of acoustic signals from biological noise associated with increased nocturnal activity of crustaceans and other marine organisms. We accounted for this observed daily bias in the array's detection capability by combining detections into hour bins; we observed similar transmitter detection rates once the data were filtered and analyzed at the scale of hours. By standardizing the detections of blue rockfish using hours as an indication of presence or absence rather than individual detections, we are confident that differences in detections are due to actual movements of fish and not due to characteristics of the acoustic environment.

## Conclusions

Blue, black, and olive rockfishes successfully cohabitate in the kelp forest ecosystem but have distinctly different movement patterns. All three rockfishes generally have small home ranges, but different temporal activity and site fidelity to a given location. Olive rockfish maintain high site fidelity and small home ranges year round, and are diurnally active. Black rockfish are nocturnally active, have small home ranges when in residence, and demonstrate the capacity for long-distance migrations [10]. Blue rockfish are diurnally active, display small home ranges, but exhibit local migrations on a scale intermediate to the olive and black rockfish. The abundance of blue rockfish in nearshore kelp systems has been hypothesized to be due to the species’ successful exploitation of a unique niche within the kelp forest [3], [26]; our 15-month study of blue rockfish movements supports these findings.
